# Intervention based on BASNEF model increases exclusive breastfeeding in preterm infants in Iran: a randomized controlled trial

**DOI:** 10.1186/s13006-016-0089-2

**Published:** 2016-11-14

**Authors:** Sheler Ahmadi, Farideh Kazemi, Seyedeh Zahra Masoumi, Parisa Parsa, Ghodratollah Roshanaei

**Affiliations:** 1 Department of Midwifery, School of Nursing and Midwifery, Hamadan University of Medical Sciences, Hamadan, Iran; 2 Department of Midwifery &Reproductive Health, School of Nursing & Midwifery, Shahid Beheshti University of Medical Sciences, Tehran, Iran; 3 Research Center for Child & Maternity Care, Midwifery Department, School of Nursing and Midwifery, Hamadan University of Medical Sciences, Hamadan, Iran; 4 Chronic Disease Research Center, Midwifery Department, School of Nursing and Midwifery, Hamadan University of Medical Sciences, Hamadan, Iran; 5 Department of Biostatistics and Epidemiology, School of Health, Hamadan University of Medical Sciences, Hamadan, Iran

**Keywords:** Exclusive breastfeeding, Premature infant, Counselling

## Abstract

**Background:**

The objective of this study is to determine the effect of a consultation model, Beliefs, Attitudes, Subjective Norms and Enabling Factors (BASNEF), and the counselling steps using GATHER-Greet clients, Ask clients about themselves, Tell clients about their choices, Help clients choose, Explain what to do, and Return for follow-up-on the continuation rates of exclusive breastfeeding in mothers of premature infants.

**Methods:**

This is a randomized controlled clinical trial carried out on 124 mothers with premature infants hospitalized in Fatemieh Hospital, city of Hamadan, in 2014. Participants were randomly assigned to either the intervention or control groups. The initial demographic questionnaire carried out in both groups included three questions about the continuation of exclusive breastfeeding, BASNEF, a checklist related to the lactation performance documented by mothers and the weight gain of their infants. Five breastfeeding consultation sessions based on the BASNEF model and counselling steps using GATHER, were held for the mothers in the intervention group for five consecutive days. Then follow-up weight gain and the questionnaire completion were performed in both groups at 1, 2, 3 and 4 months after the intervention.

**Results:**

Baseline characteristics were similar in the two groups. There were no significant differences between both groups in the rate of exclusive breastfeeding, lactation performance and infant weight at baseline. The intervention group had significantly higher rates of exclusive breastfeeding, 72.6% versus the control group of 16.1%, at the end of the 4 month follow-up. Also the intervention group had significantly higher mean scores of lactation performance (8.62 ± 2.08 vs 6.40 ± 1.84 in the control group) and infant weight (5694.80 ± 779.43 vs 4760.17 ± 859.12 in the control group) at the end of the 4 month follow-up.

**Conclusion:**

Breastfeeding consultation of mothers based on the BASNEF model and using GATHER counselling steps increased the rate of exclusive breastfeeding, lactation performance and weight gain of premature infants. Therefore, breastfeeding counselling sessions are recommended for all mothers of premature infants.

**Trial registration:**

Iranian Registry of Clinical Trials number IRCT2014111013405N6 and date registered, January 5, 2015.

## Background

Infants born alive before complete 37 weeks since the first day of the last menstrual period are called preterm [1]. Unfortunately, despite all the comprehensive attempts for preventing preterm labor and the birth of preterm infants, the rate is still high. On average, 9.6% of infants are born preterm worldwide [2] and in Iran the rate is 5.6–13.4% [3]. The probability of medical complications in preterm infants is four times more than that of term infants [4].

Breastfeeding is the best nutrition for supporting the suitable growth, especially brain growth, in all infants. Researchers have found that neural development is higher among the preterm infants fed on their mothers’ milk [5]. During recent years, mothers’ awareness about the advantages of breastfeeding has increased and most have selected to breastfeed their children. Discontinuing breastfeeding could impose irreparable physical, mental and socioeconomic damage to the society [6, 7]. Despite the highly valuable role of breastfeeding in preventing diseases and infection, unfortunately, preterm infants are more likely to be deprived of breastfeeding [8]. In most cases, replacing breastfeeding with artificial nutrition has been the mothers’ decision with concerns about their child’s weakness, lack of milk supply, and hunger, crying and unsettled children, and refusal to take the breast [9].

Exclusive breastfeeding means feeding infants only on mothers’ milk without receiving other liquids and solid foods, except vitamins, minerals, and medicine. The continuation of exclusive breastfeeding occurs when infants are exclusively fed on mothers’ milk during the first 4–6 months after their birth [10]. Data from Disease Control and Prevention Center in 2012 in the USA have reported the prevalence of exclusive feeding until the sixth month as 21.9% [11]. The Iran Ministry of Health and Medical Education has considered the promotion of breastfeeding as one of the important strategies for the growth and survival of children and has taken effective steps in this regard [12]. Nevertheless, the prevalence of exclusive breastfeeding in the first 6 months after birth in Iran ranges from 13 to 58% [13–17]. Exclusive breastfeeding for the first 6 months can prevent 13% of deaths among under 5 year old children [18]. The World Health Organization has recommended that preterm infants be exclusively fed on their mothers’ milk for 6 months. With the continuation of exclusive breastfeeding, weight gain and head circumference growth will be almost similar to those of term infants [19].

Many factors affect the continuation of exclusive breastfeeding, which include interventions during labour, medical disorders of mothers and children, anatomical anomalies of the breast, lack of skin to skin contact between a mother and her infant, mothers’ self-confidence, postpartum depression, employment, and social support [20, 21].

Additionally, the results of some studies have demonstrated that mothers’ attitude about breastfeeding, support of social networks, and existence of suitable conditions for breastfeeding in the society are among the effective factors for successful breastfeeding in the first 3 months after childbirth [22]. Mothers of premature infants need more attention and support, because the birth of these infants is very stressful [23]. This can explain the mothers’ needs for consultation and counselling support for the continuation of exclusive breastfeeding in the postpartum period. Breastfeeding counselling for the mothers of these infants must start during the hospitalization period, and must be followed up to address any problems during the breastfeeding period [24]. Behaviour study models can be effective with people’s approach toward healthy behaviours. Educational theories and models are responsible for defining and detecting the obstacles in behavioural change and for their adjustment with existing socio-cultural contexts. BASNEF, an acronym for Beliefs, Attitudes, Subjective Norms and Enabling Factors, is a comprehensive and complete model which is adopted to study behaviors, offers plans for change and defines the factors effective for the individuals’ decision making [25]. In this study, it is assumed that using a breastfeeding consultation based on BASNEF model would improve a mothers’ awareness in terms of promoting their attitude about the importance of breastfeeding for their premature infants. Also, it could improve the rate of exclusive feeding and the breastfeeding behaviors of the mothers having premature infants with the use of enabling factors such as enough information, providing the health staff for consultation and cooperation, and identification of abstract norms among mothers such as family members, mother, spouse, and health staff. Therefore, the objectives of this study were to determine the effect of counselling on different structures of BASENF model, continuation of exclusive breastfeeding, lactation performance, and weight gain in infants.

## Methods

This controlled randomized clinical trial was carried out from November 2014 to the end of June 2015 with infants and the Neonatal Intensive Care Unit (NICU) wards of Fatemieh Hospital, city of Hamadan. The primary outcome of this study was to establish the exclusive breastfeeding continuation rate and the secondary outcomes were lactation performance and weight gain of infants during the follow-up.

This research project was approved by the Ethics Committee of Research Deputy of Hamadan University of Medical Sciences with the number of 9310235220 and registered in Iranian Registry of Clinical Trials (IRCT) with ID: IRCT2014111013405N6, January 5, 2015. All participants were informed about the purpose of the study, the right to withdraw from the study at any stage without being penalized. Also, those who agreed to participate in the study signed the written informed consent form.

Before starting the study, allocation sequence was determined using 4-part randomized blocking and computerized random number table by one member of the research team, who was not involved in the selection of the samples. Accordingly, the participants were randomly selected and with the ratio of 1:1 assigned to either the intervention or control group. Codes related to each participant were placed inside opaque envelopes in the packages in order to blind the allocations. Therefore, the persons were allocated to the intervention and control groups based on the determined sequence. It is necessary to mention that, in this study, only those who collecting and analyzing the data were blind to the intervention type allocated to the groups.

The inclusion criteria included literacy, lack of addiction, lack of mothers’ suffering from underlying diseases including gestational diabetes and preeclampsia, a singleton pregnancy, a labor and birth during the 34-37th weeks of pregnancy, low birth weight infants (2000 to 2500 g), residency of the studied NIC units in Hamadan, no usage of antidepressants and psychotropic substances, no participation in breastfeeding training classes during pregnancy, and lack of anomalies in the infants. The exclusion criteria were stopping breastfeeding at the discretion of physicians, existence of infectious diseases in the breast, existence of metabolic diseases in infants, infants' death for any reason during the study, and birth of infants suffering from severe respiratory distress and hospital infection, which requires nothing to be taken by mouth (NPO).

Data collection tools included the questionnaire of demographic characteristics and pregnancy information, BASNEF questionnaire, checklist of observation and counselling regarding lactation performance, and an infant weighing scale. The demographic characteristics were studied by a researcher-made questionnaire which included questions about personal characteristics, pregnancy records, continuation of exclusive breastfeeding, and questions related to the present pregnancy. To evaluate the continuation of exclusive breastfeeding, three questions were used including the frequency of use of fluids (other than breast milk), frequency of use of solids (other than breast milk), and frequency of use of formula instead of mothers’ milk.

To evaluate the lactation performance in the mothers, the checklist used by Shahnazi [26] was applied before intervention and during the months of follow-up. This checklist included 17 questions about the signs of correct breastfeeding (e.g., baby’s body is in close contact with the mother’s body, baby’s head and body are in one direction, and the majority of areola is in the baby’s mouth etc.). The questions were answered either “yes” or “no”. An score of “1” was given to the positive answers, and “0” to the negative responses. Thus, the maximum score was 17 and the minimum score is zero.

The initial questionnaire based on the structures of attitude, enabling factors, and abstract norms in BASNEF model, and the questionnaire used by Shahnazi [26] was applied. This included the structures of awareness, behavioural intention, and evaluation of results in BASNEF model, the questionnaire used in Masoumi’s work [27] was employed. The final formulated questionnaire had 6 parts: awareness of breastfeeding (related to the structures of individual beliefs with 20 three-option questions; the correct response had a score of 1 and wrong as well as no responses scored 0), attitude (related to individual beliefs with 10 five-option questions, in which score range was between 4 as the most desirable response and 0), enabling factors (7 four-option questions, in which the correct response had score of 1 and wrong as well as no responses scored 0), abstract norms (8 questions with the score range of 1 to 3), behavioural intention (3 multiple-choice questions, in which the correct response scored 1 and wrong as well as no responses scored 0), and evaluation of the results (5 multiple-choice questions, in which the correct response scored 1 and wrong as well as no responses scored 0). Therefore, scoring was within 0–20, 0–40, 0–7, 8–24, 0–5, and 0–3 for awareness, attitude, enabling factors, abstract norms, evaluation of results, and behavioural intention, respectively. The difference in the number of questions for different structures differed and were calculated based on 100 to facilitate the comparison of scores.

**Fig. 1:**
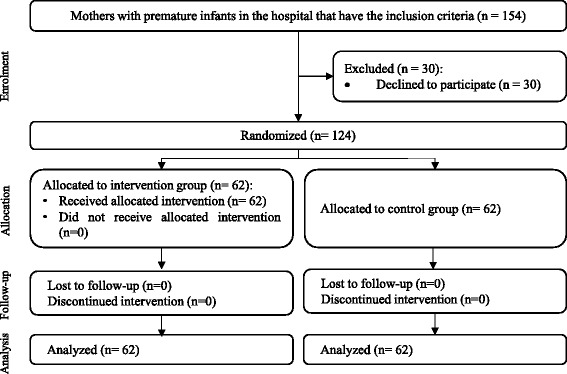
Flow diagram of participants

To determine the validity of the used questionnaires in the study, content validity method was used. Thus, the questionnaires were studied by six related experts (three in reproductive health, one in health education, and two in midwifery) from the School of Nursing and Midwifery, Hamadan University of Medical Sciences, and the necessary modifications were applied. To determine the reliability, a pilot study was carried out on 20 qualified persons. Cronbach’s alpha was calculated in BASNEF questionnaire for the questions related to awareness (α = 0.72), attitude (α = 0.70), abstract norms (α = 0.9), enabling factors (α = 0.83), evaluation of results (α = 0.79), and behavioural intention (α = 0.86). Cronbach’s alpha was also calculated for the questions related to the continuation of exclusive breastfeeding (α = 0.81) and checklist of observation and consultation of lactation performance (α = 0.92). In order to determine the reliability of the infant weighing scale, its reliability was controlled and confirmed by a standard 500 g weight before each time of weight measurement. For recruitment, the researcher visited the infants and NICU wards of Fatemieh Hospital, city of Hamadan, on a daily basis. After stating the research objectives and getting the written consent letter from the mothers having the inclusion criteria, they were selected for the participation in the study (Fig. 1). It is necessary to mention that the time of starting breastfeeding counselling was from the start of infants’ oral nutrition with the verification of the consulting doctor until their discharge from the hospital. Before starting the counselling and then once per month for 4 months, all of the infants were weighed by a standard scale and wearing a diaper only. The questionnaires were filled out in person and face-to-face before the intervention and then with monthly follow-up for 4 months after the birth for both the intervention and control groups in the hospital. For the mothers of the intervention group, five breastfeeding counselling sessions were held individually and face-to-face by the researcher, which lasted for 30 min and on consecutive days and were based on the counselling steps of the reproductive health services GATHER (using model, pamphlets, and incentive packages) and daily objectives table. GATHER counseling steps including, greet clients, ask clients about themselves, tell clients about their choices, help clients choose, explain what to do, and return for follow-up [28]. For the premature infants who were discharged earlier from the hospital, the rest of the counselling sessions were held in coordination with the parents and arranged appointments in the hospital (Table 1). At the end of the 4 month, an incentive package including a book on the nutrition of premature infants and training Compact Disc (CD) was given to the mothers in both groups. Further, in one of the sessions, one of the relatives of the mothers with maximum participation in their decision making was present and received the necessary training. It is necessary to mention that the mothers of the control group only received the conventional trainings by the staff of infants ward in the postpartum period.

**Table 1:** Breastfeeding training program

First session
Objective: • Consultation and counselling to get mothers familiar with methods of breastfeeding premature infants Training content: • Counselling about different breastfeeding methods • Defining natural and artificial breastfeeding Second session Objective: • Counselling to get mothers familiar with several conventional definitions and explanations in breastfeeding Training content: • Defining exclusive breastfeeding • Reasons for breast infection in the postpartum period • Reasons for infants’ diarrhoea in artificial nutrition Third session Objective: • Counselling to get mothers familiar with the advantages and disadvantages of natural and artificial feeding of premature infants Training content: • Advantages of natural breastfeeding • Wrong reasons for starting artificial breastfeeding by mothers and describing its complications Fourth session Objective: • Counselling to get mothers familiar with correct techniques of breastfeeding in premature infants Training content: • Reasons for using natural breastfeeding • Mentioning correct breastfeeding techniques, explaining them, and correctly implementing them by mothers Fifth session Objective: • Review of the content and conclusion Training content: • Reasons for using natural breastfeeding • Mentioning correct breastfeeding techniques, explaining them, and correctly implementing them by mothers

Based on the information obtained from the article by Arzani et al. [29], in which the rates of breastfeeding in case and control groups were equal to 60 and 40%, considering α = 0.05, power = 80% and calculating 10% loss of participants, the number of participants needed per group was 62 persons.

$$ n=\frac{{\left({Z}_{1-\frac{\alpha }{2}}\ \sqrt{\overline{P}\left(1-\overline{P}\right)}+{Z}_{1-\beta}\sqrt{P_1\left(1-{P}_1\right)+{P}_2\left(1-{P}_2\right)\ }\ \right)}^2}{{\left({P}_1-{P}_2\right)}^2} $$

Data were analyzed in SPSS21. For the normality analysis of the data, Kolmogorov-Smirnov test was used. To explain the personal characteristics of the participants, descriptive statistics (number, percentage, mean, and standard deviation), *t*-test, Fisher’s Exact Test, and Chi-square test were used. Repeated Measures ANOVA test, Bonferroni’s post-hoc test, and independent *t*-test were used to compare the mean scores in different structures of BASNEF model, lactation performance, and the weight gain of infants during the follow-up. To compare the continuation of exclusive breastfeeding during the follow-up months Mann-Whitney and Chi-square tests were used. In the data analysis, *p* < 0.05 was considered significant.

## Results

### Baseline characteristics

In this study, there were 62 mothers in each group. Mean age of mothers in the intervention and control groups were 27.13 ± 0.53 and 27.19 ± 0.60 respectively. More than 90% of mothers were housewives and less than a quarter of mothers in each group were nulliparous. 80.65% of women in intervention group and 72.58% in control group had breastfeeding experience. Other details are indicated in Table 2. No significant difference was observed between the two groups in terms of the studied variables.

**Table 2:** Demographic and obstetric characteristics of participants

	Intervention group^a^ *n* = 62	Control group^a^ *n* = 62	Statistic	*p* -value
Age of mother, years	27.13 ± 0.53	27.19 ± 0.60	*t* = 1.54	0.52^b^
Education level				
Illiterate and elementary	21 (33.8)	21 (33.8)	*χ* ^2^ = 0.23	0.99
Middle	14 (22.6)	15 (24.2)		
Diploma	14 (22.6)	15 (24.2)		
Academic	13 (21.0)	11 (17.8)		
Occupation				
Housewife	56 (90.3)	59 (95.2)	*χ* ^2^ = 3.07	0.21^c^
Employed	6 (9.7)	3 (4.8)		
Baby gender				
Male	33 (53.2)	33 (53.2)	*χ* ^2^ = 0.001	1.0
Female	29 (46.8)	29 (46.8)		
Type of delivery				
Normal vaginal delivery	19 (30.6)	19 (30.6)	*χ* ^2^ = 0.001	1.0
Caesarean section	43 (69.4)	43 (69.4)		
Breastfeeding history				
Yes	50 (80.65)	45 (72.58)	*χ* ^2^ = 1.66	0.43
No	12 (19.35)	17 (27.42)		
Duration of previous breastfeeding, months	6.81 ± 0.53	6.57 ± 0.74	*t* = 0.83	0.35^b^
Number of previous children				
0	14 (22.58)	15 (24.19)	*χ* ^2^ = 0.34	0.98
1	31 (50.0)	30 (48.39)		
2	12 (19.35)	12 (19.35)		
≥ 3	5 (8.07)	5 (8.07)		
Infant feeding of previous children				
Breast milk	48 (77.42)	45 (72.58)	*χ* ^2^ = 2.93	0.40^c^
Formula	12 (19.35)	13 (20.97)		
Cow milk, etc.	2 (3.23)	4 (6.45)		
History of exclusive breastfeeding with the last baby				
Yes	42 (67.74)	45 (72.58)	*χ* ^2^ = 2.43	0.49
No	20 (32.26)	17 (27.42)		

^a^Number (%), mean ± SD, ^b^ *t*-test, ^c^Fisher’s Exact Test, the remainder Chi-square test

### Different structures of BASNEF model

To compare the mean scores in different structures of BASNEF model (except behavioral intention) during the study in both groups, Repeated Measures ANOVA test was used. Findings showed the intervention group had significantly higher rates of mean scores in the structures of the model at 4 months, versus controls (*p* < 0.001). Bonferroni’s post-hoc test demonstrated that the intervention group had significantly higher rates of mean scores in the structures of the model at 4 months, versus controls (1, 2, 3, and 4 months after the intervention) (*p* < 0.001). There were no significant differences between the mean scores of both groups in the structures at the pretest stage; however, this mean showed a statistically significant difference at the stages of 1, 2, 3, and 4 months after the intervention. Such a difference existed in all the structures of the model (*p* < 0.001) (Table 3). To study the behavioural intention, three questions were used: “What kind of feeding do you select (breastfeeding or dried milk)? Do you intend to continue exclusive breastfeeding of your child for 6 months? Do you want to continue breastfeeding for 2 years?” Comparison of the control and intervention groups by Chi-square test at the pretest stage showed no significant difference between the two groups in any of the questions; but, comparison of the two groups in different months of follow-up demonstrated the intervention group had significantly higher rates for all the three questions between the two groups (*p* < 0.001).

**Table 3:** Comparison of the mean scores of structures in BASNEF model

		Baseline	First month after Intervention	Second month after Intervention	Third Month after Intervention	Fourth Month after Intervention	RM^a^ between groups
Knowledge	Intervention group	47.42 ± 12.43	67.10 ± 12.10	63.47 ± 12.03	62.30 ± 12.66	60.2 ± 12.60	F = 16.30 *p* < 0.001
	Control group	47.30 ± 9.70	44.40 ± 10.9	39.90 ± 9.70	37.90 ± 9.10	37.50 ± 8.70	
Attitude	Intervention group	34.10 ± 4.55	40.10 ± 2.40	43.20 ± 2.40	44.90 ± 2.80	45.30 ± 3.50	F = 5.60 *p* < 0.001
	Control group	33.30 ± 4.50	33.30 ± 4.20	33.20 ± 4.50	33.00 ± 5.20	33.20 ± 5.20	
Subjective norms	Intervention group	13.65 ± 1.79	13.67 ± 1.30	13.82 ± 1.62	13.90 ± 1.39	14.11 ± 1.55	F = 39.32 *p* < 0.001
	Control group	13.62 ± 1.37	12.26 ± 0.75	12.25 ± 0.83	12.21 ± 0.53	12.18 ± 0.52	
Enabling factors	Intervention group	8.99 ± 1.51	10.40 ± 1.12	11.29 ± 0.76	11.39 ± 0.78	11.48 ± 0.71	F = 324.14 *p* < 0.001
	Control group	8.04 ± 1.69	7.71 ± 1.02	7.43 ± 1.02	7.51 ± 1.17	7.52 ± 1.12	
Outcome evaluation	Intervention group	2.05 ± 1.19	3.38 ± 1.10	3.17 ± 1.00	3.05 ± 1.17	3.05 ± 1.77	F = 39.42 *p* < 0.001
	Control group	2.02 ± 1.13	2.48 ± 1.12	1.87 ± 1.13	1.56 ± 1.12	1.27 ± 1.06	

^a^Repeated Measures ANOVA test

### Continuation of exclusive breastfeeding

Comparison of the two groups in terms of continuation of exclusive breastfeeding showed that there was no statistical significant difference between the two groups at the pretest stage (53.2% in intervention group versus 48.4% in control group). After training, the intervention group had significantly higher rates of exclusive breastfeeding in the first, second, third, and fourth months versus controls (*p* < 0.001) (Table 4).

**Table 4:** Comparison of the continuation of exclusive breastfeeding during the follow-up months

Exclusive breastfeeding		Baseline	First month after intervention	Second month after intervention	Third month after intervention	Fourth month after intervention
		*n* (%)	*n* (%)	*n* (%)	*n* (%)	*n* (%)
Intervention group *n* = 62	yes	33 (53.2)	48 (77.4)	44 (71.0)	38 (61.3)	45 (72.6)
	no	29 (46.8)	14 (22.6)	18 (29.0)	24 (38.7)	17 (27.4)
Control group *n* = 62	yes	30 (48.4)	4 (6.5)	4 (6.5)	3 (4.8)	10 (16.1)
	no	32 (51.6)	58 (93.5)	58 (93.5)	59 (95.2)	52 (83.9)
*p* ^a^		0.59	<0.001	<0.001	<0.001	<0.001

^a^Chi-square test

### Lactation performance and weight gain

To compare lactation performance and weight gain of infants during the follow-up in both groups, the test Repeated Measures ANOVA was used. As observed in Table 5, the intervention group had significantly higher mean scores of the lactation performance and weight gain in the infants at 4 months compared to the control group (*p* < 0.001). Bonferroni’s post-hoc test showed the intervention group, versus control group, had significantly higher rates in the mean comparison of scores in terms of lactation performance and weight gain of infants between 1, 2, 3, and 4 months after the intervention and pretest stages (*p* < 0.001). Comparing the mean of the intervention and control groups using independent *t*-test showed no significant difference at the baseline, whereas the intervention group had significantly higher rates during 1, 2, 3, and 4 months after the intervention compared to the control group (*p* < 0.001).

**Table 5:** Comparison of the mean scores of infant weight and lactation performance during follow-up in both groups

		Baseline	First month after intervention	Second month after intervention	Third month after intervention	Fourth month after intervention	RM^a^ between groups
Infant weight	Intervention group	2112.26 ± 439.22	2818.62 ± 588.54	3844.83 ± 642.68	4743.10 ± 678.78	5694.80 ± 779.43	F = 22.84 *p* < 0.001
	Control group	2126.29 ± 453.96	2422.58 ± 607.18	3296.45 ± 684.24	4145.08 ± 749.60	4760.17 ± 859.12	
	*p*	0.86	<0.001	<0.001	<0.001	<0.001	
Lactation performance	Intervention group	5.61 ± 1.86	7.52 ± 2.21	8.01 ± 1.88	8.70 ± 1.93	8.62 ± 2.08	F = 19.05 *p* < 0.001
	Control group	5.85 ± 1.84	5.90 ± 1.96	5.84 ± 1.84	6.38 ± 2.30	6.40 ± 1.84	
	*p*	0.07	<0.001	<0.001	<0.001	<0.001	

^a^Repeated Measures ANOVA test

## Discussion

The results of this study showed that the intervention group had significantly higher rates of the continuation of exclusive breastfeeding at months 1, 2, 3, and 4 (vs controls). The exclusive breastfeeding rate was 72.6% in the intervention group and 16.1% in the control group at the end of 4 months. Also, according to the study, the mothers in the control group used more formula to feed their babies than the mothers in the intervention group. Some studies have shown that exclusive breastfeeding has a high correlation with the growth of premature infants [30, 31]. Furthermore, the results show that the frequency and severity of infectious diseases including bacterial meningitis, diarrhoea and respiratory tract infections, in infants fed with breast milk is less than infants fed with formula [32].

Findings of the present study were similar to those by Arzani et al. [29], in which they could increase the continuation of exclusive breastfeeding in low-weight infants and improve lactation performance in 55 mothers of such infants during the first 3 months after their birth. Takour et al. [33] showed that such trainings resulted in a 60% increase in the rate of exclusive breastfeeding and 40% decrease in respiratory diseases, diarrhoea, and infection in low-weight infants of the intervention group compared with the control group, at the end of 2 months. Additionally, Aidam et al. [34] showed that breastfeeding consultation during pregnancy and after birth could increase the exclusive breastfeeding rates in term infants during the first 6 months.

Among other findings of the present study was the improvement of lactation performance during the follow-up period in the intervention group. Results of many studies have shown that active and direct participation of professional care-providers such as midwives in the breastfeeding training and consultation of mothers can have highly positive effects on the increase of exclusive breastfeeding process in term and preterm infants and its improvement [35–39]. Based on Ahmed [40], breastfeeding training for the support of mothers having premature infants during the 3 months of follow-up can improve lactation performance and awareness of mothers about feeding infants on their milk. Rea et al. found a statistically significant difference between lactation performance of mothers before and after breastfeeding consultations [41]. Aghababaee et al. [42] showed that trainings for feeding infants on mothers’ milk could remarkably improve lactation performance in the mothers who have given birth to their first children; these results were compatible with those obtained from the present study.

The findings of our research showed that most mothers were aware about feeding their milk to their infants. But, this awareness is mostly about the advantages and importance of breastfeeding for mothers and children. Awareness of mothers about the practical problems that may stand in the way of successful breastfeeding premature infants is still limited and there are no effective or logical solutions to prevent such problems. These findings were in agreement with the results obtained by Dewan et al. [43] about the awareness of adolescent mothers of feeding their milk to their baby. The study by Shahnazi et al. [26] showed that breastfeeding training, based on the BASNEF model can increase the awareness of mothers. Mokhtari et al. [44], Aghababaie et al. [42], and Roby et al. [45] have demonstrated that using training programs results in a significant increase of mothers’ awareness in terms of breastfeeding. Also, Scoot et al. [46] found a significant positive relationship between the continuation of feeding mothers’ milk and level of their awareness. These results were in agreement with the findings of the present work.

The aim of breastfeeding consultation and counselling for mothers is not only to increase breastfeeding knowledge and skills, but also to change mothers’ attitude about breastfeeding [47]. Results obtained from the present study showed the positive effects on the breastfeeding attitude of women; such results were in line with the findings by Mosafaye Khomamias [48], and Masoumi et al. [27]. However, it was not compatible with the studies carried out by Charkazi et al. [49], Alaei et al. [50], and Ghaffari et al. [51], who have reported higher baseline scores for attitudes about breastfeeding. This difference can be related to different cultural fields, time interval with the present study, and using different tools in the study. Based on Charkazi’s work [49], mothers with more desirable attitudes had better lactation performances. Salehi believes that breastfeeding training programs compatible with mothers’ cultures in their society can result in greater changes of attitudes and beliefs of mothers about exclusive breastfeeding [52].

Studies have demonstrated that mothers who have been supported by relatives and peers have stopped exclusive feeding on mothers’ milk by less than 30% compared to other mothers [53–55]. Furthermore, mothers who feel that when society highly considers a mothers role, it improves the breastfeeding role [56]. Giles et al. [57] highlighted the importance of the supportive role of mothers, spouses, close friends, and those employed in medical professions regarding the continuation of breastfeeding, and found that mothers of breastfeeding women have a more important role in terms of starting and continuing breastfeeding. Also demonstrated was the training and consultation of doctors and midwives are important in this regard. Britni et al. showed that support by spouses and accepting the breastfeeding behaviour as a social norm, support by friends, participation in counselling classes, and postpartum support and follow-up are the most important factors for starting and continuing breastfeeding [23]. In Sharifirad’s investigation [26], the results showed no significant difference (except health staff), between mothers’ abstract norms before and after the intervention; i.e., training mothers by itself did not have a great role in changing the patterns and norms related to the correct breastfeeding behavior. Also, for changing norms, there is a need for a more extensive training program which could also involve the relatives of mothers such as spouse, mother, mother-in-law, and friends. In the present study, we tried to overcome this limitation, therefore, during the consultation sessions based on abstract normative structure before the test, we held a face-to-face training class for the one person with maximum score and the highest role in mothers’ decision-making. In conclusion, in our study, the intervention group had significantly higher rates of mean scores in the abstract norms at 4 months, versus the controls (1, 2, 3, and 4 months after the intervention).

In the present study, the existence of social support by health staff was studied by using the enabling factors from the BASNEF model. Based on the study by Daly et al. [58], counselling and support received by the family and society are among the most important enabling factors for breastfeeding facilitation. Health staff can play an important role in helping mothers and their families solve breastfeeding problems. They can provide real and valuable information and empathetic support for mothers. The presence of trained and active health staff is a component of the much needed social support for the continuation of exclusive breastfeeding [59]. In the work by Shakespeare et al. [60], mothers mentioned the inaccessibility of the health and medical staff is a problem in the continuation of exclusive breastfeeding. Furthermore, in most of the investigations, mothers have said that during breastfeeding, they have not been supported by health staff and could not access them when problems arose [61]. Lewis et al. [62] believed that getting information via training classes during pregnancy and after birth, and that magazines and TV are also effective in starting and continuing exclusive breastfeeding.

This study had some limitations, including failure to fill the questionnaire completely by some of the mothers as some questions remain unanswered, and difficulty obtaining breastfeeding information by some mothers from other centers and clinics during follow-up. To overcome some of these limitations, mothers received explanations about the impact of their answers to the questionnaire in the study results.

## Conclusions

Findings of this research showed that consultation and counselling improves the continuation rate of exclusive breastfeeding for premature infants, decreases the need for using other liquids, and feeding on foods other than mother’s milk, especially dried milk powder, during the first 4 months.

Additionally, it can improve the lactation performance of mothers and increase the rate of weight gain in premature infants during the first 4 months after birth. Breastfeeding consultations and counselling improves awareness, attitude, abstract norms, enabling factors, evaluation of results, and behavioural intention in the intervention group mothers with premature infants compared with the control group ones during the first 4 months.

## Abbreviations

BASNEF: Beliefs, Attitudes, Subjective Norms and Enabling Factors
CD: Compact Disc
GATHER: Great clients, Ask clients about themselves, Tell clients about their choices, Help clients choose, Explain what to do, and Return for follow-up
IRCT: Iranian Registry of Clinical Trials
NICU: Neonatal Intensive Care Unit
NPO: Nothing by mouth
